# Effect of Hydrocolloid Gums on the Pasting, Thermal, Rheological and Textural Properties of Chickpea Starch

**DOI:** 10.3390/foods8120687

**Published:** 2019-12-16

**Authors:** Syed Ali Shahzad, Shahzad Hussain, Abdellatif A. Mohamed, Mohamed S. Alamri, Mohamed A. Ibraheem, Akram A. Abdo Qasem

**Affiliations:** Department of Food Sciences, College of Food and Agricultural Sciences, King Saud University, P.O. Box 2460, Riyadh 11451, Saudi Arabia; abdmohamed@ksu.edu.sa (A.A.M.); msalamri@ksu.edu.sa (M.S.A.); mfadol@ksu.edu.sa (M.A.I.); aqasem@ksu.edu.sa (A.A.A.Q.)

**Keywords:** chickpea, hydrocolloids, gums, rheology

## Abstract

The study was planned to evaluate the effect of non-commercial gums as compared to commercial gums. The concentration dependent effect of two commercial (arabic, xanthan) and four non-commercial (cress seed, fenugreek, flaxseed, okra) polysaccharide gums on the pasting, rheological, textural and thermal properties of chickpea were investigated by rapid visco analyzer (RVA), hybrid rheometer, texture analyzer and differential scanning calorimetry (DSC). Blends were prepared by replacing chickpea starch at 0.5% and 2.0% with gums, whereas native chickpea starch was used as a control. RVA data showed that peak and final viscosities were dramatically increased with xanthan contrary to reduction with gum arabic, flaxseed and okra gums. Hybrid rheometer displayed that storage and loss moduli were increased as a function of angular frequency and dominance of elastic properties over viscous ones. Xanthan blend was less temperature dependent due to dramatic decrease in activation energy value as compared to control while other gums were more temperature dependent. The magnitude of this effect was reliant on the type and concentration of gum. After storage for 21 days at −20 °C, total syneresis was reduced with the incorporation of xanthan and cress seed and also with high levels of gum arabic, flaxseed and fenugreek gums. The gel hardness was increased after overnight storage at ambient temperature (23 °C) with fenugreek while reduction in hardness was observed with xanthan, flaxseed and okra gums. The presence of gums resulted in significantly higher onset and peak temperatures determined through differential scanning calorimetry.

## 1. Introduction

Hydrocolloids are high molecular weight biopolymers and are water-soluble due to the presence of polar groups in the chains. They form viscous dispersions when dissolved in water and act as thickening/gelling agents even at very low concentration [1]. Food industry is using them as additives to modify the texture, rheology of aqueous suspensions, appearance and to control the functional properties of different products by exploiting their inherited capability of holding water and stabilizing emulsions and dispersions [2]. Hydrocolloids or gums are used in the manufacturing of cakes, biscuits, bread, mayonnaise, ice cream, desserts, jellies and salad dressings. The functionality of these hydrocolloids is dependent on their origin, quantity used, extraction method, interactions with other ingredients, chemical structure and any type of structural modifications [3,4]. Recently, Yang et al., [5] and Shrivastava et al. [6] studied the effect of gums on the pasting, gelatinization, syneresis and textural properties of various starches and reported that gums impart promising effects on these properties.

Starches are found in seeds, fruits, leaves, and stems, roots and fruits of plants and are considered as the most abundant source of carbohydrate reserve in plants. Major sources of starch include tubers (65–85%), cereals (40–90%), roots (30–70%), legumes (25–50%) and some immature fruits (up to 70%) like mangoes or bananas [7]. Starches consist of two different polymers i.e., amylose and amylopectin. The starches are widely used in bakery, dairy, snacks, soups, sauces, gravies and meat products. They are found to affect their moisture retention, gel formation, viscosity, texture and binding.

Chickpea (*Cicer arietinum* L.) is a legume that belongs to family Fabaceae, widely cultivated in Australia, Asia, Mediterranean basin and North America [8]. The main compounds found in chickpea seeds are carbohydrates (63% by weight), of which starch is the most represented (37.5–50.8%) [9,10]. Chickpea starch is considered commercially important for many processed foods. It is used in foods because of its gelation, gelatinization and pasting properties. However, in most of the cases, the properties of native starch are not ideal, e.g., inferior gelatinization stability during prolonged heating, acidic environments or high shear behavior that cause disruption of the network of starch gel [11]. Furthermore, on cooling, starch has a great ability to retrograde, which ultimately leads to syneresis. Thus, native starches should be modified either physically or chemically by using additives that enhances their (native starches) functional properties, such as non-starch hydrocolloids [12]. Incorporation of hydrocolloids can modify both rheological and structural features like pasting properties and gelatinization temperature [13]. It also plays an important role in improving or maintaining the desirable textural characteristics and stabilizing the products over a long period of storage [14]. Thus, starch/gum blends are useful in wide range of foods products. In this study, our prime objective was to investigate the concentration dependent effect of some non-commercial gums (cress seed, fenugreek, flaxseed and okra) and commercial gums (gum arabic, xanthan) on the rheological, functional, pasting, and textural properties of chickpea starch. The results could be useful to identify natural, relatively safe and non-conventional sources of available hydrocolloids that can be used as an alternative to enzymatic and chemical modification of different starches.

## 2. Results and Discussions

### Pasting Properties of Starch Gum Blends

Pasting characteristics of chickpea starch suspensions with or without gums are summarized in Table 1. There was a significant difference in the peak viscosity of chickpea starch with different gums at both levels (0.5 and 2%). The difference was dependent on the gum type and its concentration. Peak viscosity is actually the equilibrium point between maximum granule swelling and leaching of amylose. A significant increase in the peak viscosity of chickpea starch was observed with xanthan (4256 cP and 5611 cP), followed by fenugreek gum) 4040 cP and 4311 cP) at 0.5 and 2% concentration, respectively. It was proposed that the increase in the peak viscosity was due to the association of gums with leached amylose and low molecular weight amylopectin [15]. Another possible explanation, by assuming the starch/gum/water system to be biphasic in which gums were localized in the continuous phase and its concentration was dramatically increased due to the swelling of the starch granules during gelatinization. It could have resulted in higher viscosities [15]. Previously, similar results were reported by the same author. According to Inglett et al. [16], starch blends with higher viscosities could be used in bread production for better development of texture. A significant decrease in the peak viscosity with gum arabic, cress seed and okra as compared to the control were also noticed. The plausible explanation was the ability of the gums to enclose the granules of starch and restrict their swelling during gelatinization, which resulted in reduction in the peak viscosity. Furthermore, there might be an antagonistic association between gums and amylose in continuous phase that encloses starch granules. These findings were in agreement with the previous investigations [6,17]. The association among gum and leached components primarily amylose was responsible for increase or decrease in viscosity of starch paste [18]. Starch blends with low viscosity could be used in confectionary products, weaning foods or as protective coating in different food applications [6]. Significant decrease in the breakdown viscosity for all blends was observed compared to the control, except cress seed at 0.5% gum level and at 2.0% gum level for xanthan and fenugreek, where they behaved similar to the control. The breakdown parameter indicates the extent of rupturing of the swollen starch granules, which results in loss of integrity and disruption. The process is called breakdown and viscosity is termed as breakdown viscosity. It was found lowest in the chickpea/xanthan blend (1069 cP) and chickpea/arabic blend (789 cP) at 0.5% and 2.0% gum level, respectively. Lower values of the blends than those measured for the controlled sample (1350 cP) indicated that chickpea starch granules with gums were less resistant to heating and mechanical shearing [18]. The decrease in the breakdown viscosity was ascribed to the less swelling of the granules because of the presence of the gum [19]. To ensure that decrease in the breakdown viscosity was due to the certain role of the gum, not only because of less starch. As an example, breakdown viscosity of the control was 1350 cp. Theoretically, it could be 1343 and 1323 cP for the 0.5 and 2.0% blends, respectively, but for gum arabic, it was found to be 1153 and 789 cP for 0.5 and 2.0% blends. There was a difference of 190 and 534 cP from the actual value which means the difference was not only due to the decreasing starch content in the blends as compared to the controls containing 100% starch but also due to the certain role of the gum. These assumptions are based on our previous works on the role of okra gum in rice, sorghum, potato and sweet potato starches [20,21].

In case of final viscosity, there was a dramatic increase in viscosity 6317 cP and 6760 cP with xanthan concentrations of 0.5% and 2.0%, respectively. It was might be due to the thickening effect of the xanthan that results in raise in the viscosity of continuous phase and overall suspension. Similar results were reported by Yadav et al. [22] while working with colocasia starch and xanthan gum. The substantial decrease in the final viscosity values with all other gums at both levels were detected except with fenugreek that was at 0.5% concentration (6207 cP), almost close to the control (6146 cP) but with 2.0% concentration (6330 cP) it was significantly higher than the control as shown in Table 1. Decrease in final viscosity might be attributed to the presence of less amount of starch in the blend due to the replacement with the gum [23].

Setback is an indicator of starch retrogradation and texture of starch based food products directly dependent on setback viscosity. Leached amylose molecules quickly unite during cooling of starch paste and form junction zones of amylose that were responsible for the setback [24]. Blends had a positive relationship on setback as shown by lower setback values than that for the control. It might be due to gums that acted as a barrier between leached amylose that caused a problem in lining up together and the network development [17]. To check the effect of gums on setback and to ensure the decline in setback values due to either the lower starch content or a particular role of gum, setback values were further analyzed. As per Alamri et al. [17], RVA measured values (Actual) along with the calculated values (theoretical) of setback were tabulated in Table 2. Theoretical values were estimated by deducting 0.5% and 2.0%, respectively, from actual RVA value of plain chickpea starch. These values were compared with the blend values. The difference between actual RVA value and theoretical value was considered as particular role of gum in reducing the setback value. Higher concentration of gum caused more reduction in the setback value except for fenugreek. As an example, change in the setback was only due to the reduction in the starch. Therefore, if the sample had 0.5% less starch, its setback value should be (3672 cP), but with gum arabic it was (3344 cP). This difference of 328 cP was due to the presence of 0.5% gum arabic. It means that the difference was not only due to the starch replacement but also due to the presence of gum. Pasting temperature is an indicator of minimum energy required to cook starch containing food products. It is also a point where viscosity starts increasing. It is assumed that this is the beginning point of gelatinization. It was significantly higher with all gums at both levels except for okra at 2.0% level. Leite et al. [25], who studied the effect of the xanthan gum and carrageenan on cassava starch, also observed an increase in the pasting temperature by the addition of the gums.

## 3. Dynamic Rheology

**Figure 1:**
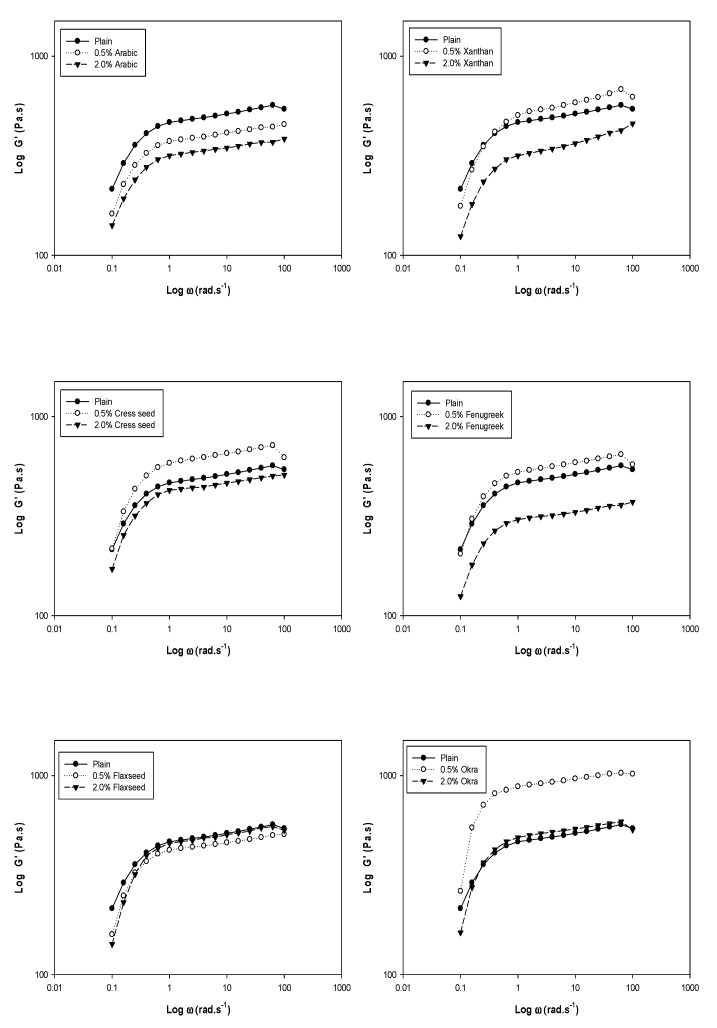
Plots of log *G*′ versus log *ω* of chickpea starch gum blends at different gum concentrations.

**Figure 2:**
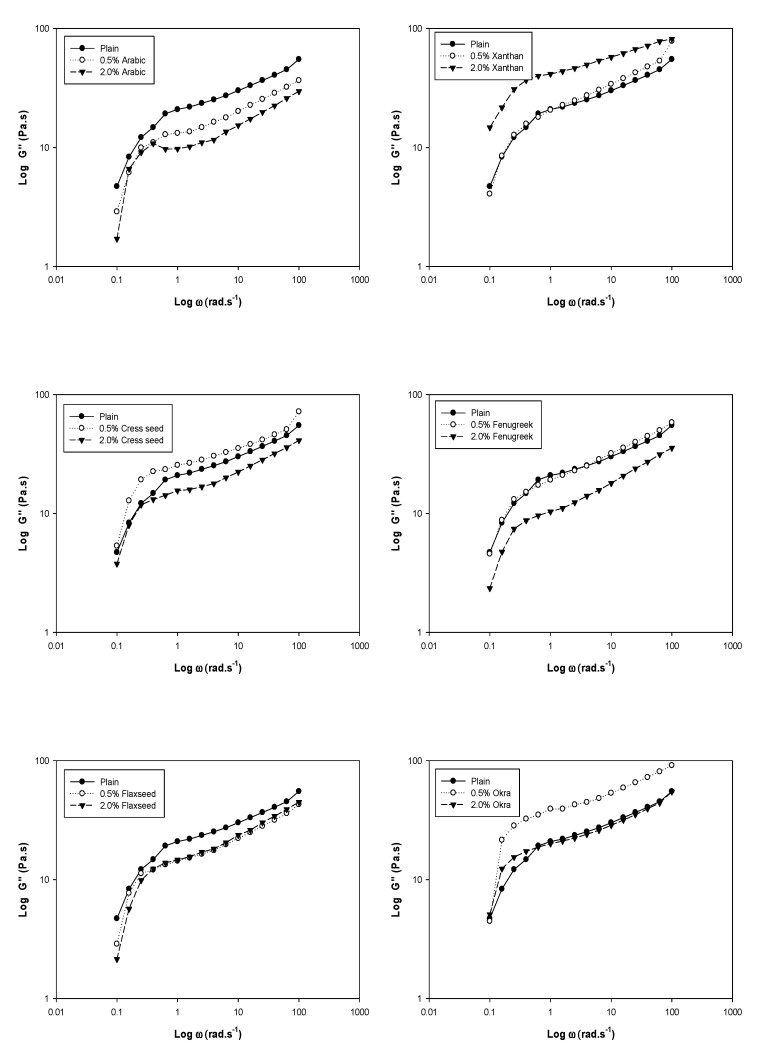
Plots of log *G*″ versus log *ω* of chickpea starch gum blends at different gum concentrations.

**Figure 3:**
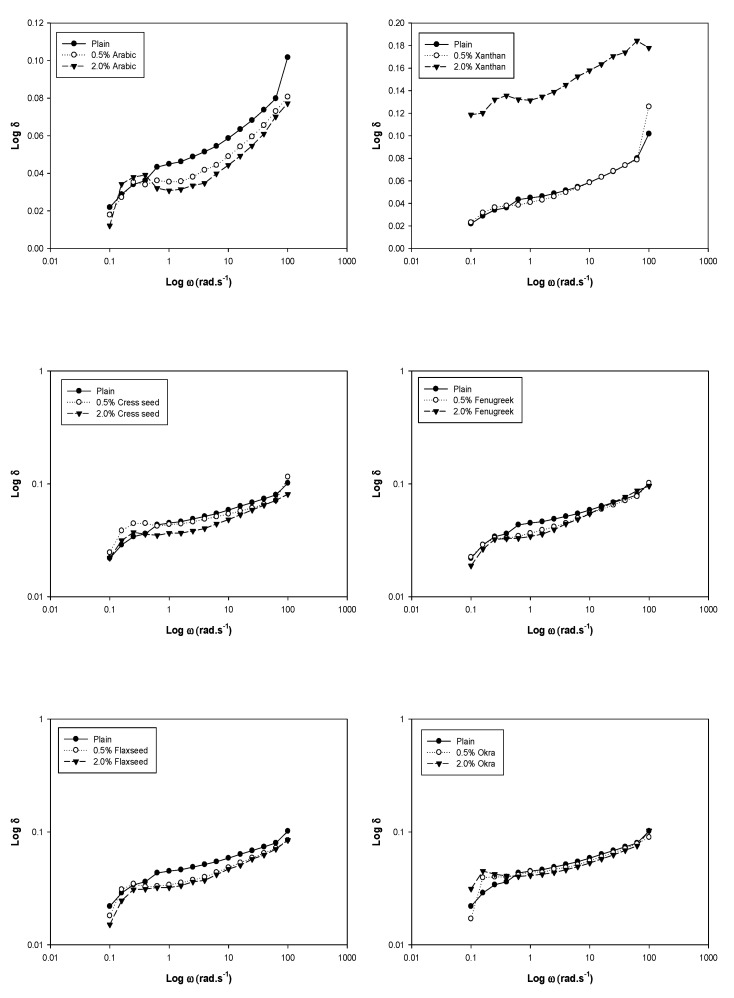
Plots of logδ versus log *ω* of chickpea starch gum blends at different gum concentrations.

The changes in storage modulus (*G*′), loss modulus (*G*″) and dynamic mechanical loss tangent (tanδ = *G*″/*G*′) of chickpea starch and the blends are shown in Figure 1, Figure 2 and Figure 3. For viscoelastic materials, *G*′ indicates the elastic character and solid like properties, while *G*″ indicates the viscous character and liquid like properties [26]. The magnitude of storage moduli, loss moduli and tanδ were increased with increasing angular frequency, and no crossovers were noticed between moduli. It was observed that storage moduli of all samples was much higher compared to the loss moduli throughout the frequency range (0.1–100 rad/sec), which reveals supremacy of elastic properties over viscous ones with largely frequency dependence at low frequencies. The large frequency dependence indicates that there was a strong association between gums and chickpea starch in the composite system. The greater the angular frequency, higher was the blend elasticity (*G*′ > *G*″). It was observed that both the plain chickpea starch and blends possessed a usual biopolymer gel network. This was factual, since the separation among the moduli was less than 0.1 except for xanthan (0.2) [27]. These results clearly revealed that gels from blends exhibited weaker consistency due to the dilution effect of starch. The effect of gum on the viscoelastic properties of the chickpea starch was variable. Blends with 0.5% gum concentration (xanthan, cress seed, fenugreek and okra) amplified the storage and loss moduli as compared to the control. This could be attributed to the raise in viscoelastic properties due to added gums. The gels get concentrated within the continuous phase of the composite system due to the available area of the phase to the gums were shrunken, because during gelatinization starch granules were swollen [28]. Thus, inclusion of gum contributed synergistically to the rheological properties of the blends. Similar results were reported by Lee and Chang [29] and Singh et al., [30], who evaluated the effect of guar, tara and locust bean gum on water chestnut and gum arabic on tapioca starch, respectively. In contrast, the magnitude of the moduli was decreased with increasing gum concentration from 0.5% to 2.0% (xanthan, cress seed, fenugreek and okra) when compared to control. The results were well supported by the observations reported for corn starch with alginate and CMC, colocasia starch with arabic and HPMC [6,31]. The decrease in the moduli could be due to a higher temperatures causing de-polymerization of macromolecules [32]. Moreover, *G*′ was decreased as a function of all types of gums concentration except flaxseed as indicated in the Figure 1. This might be due to reason that the gums at lower concentration were uniformly dispersed in the composite system. There was a synergistic effect of gum addition at low level, as both gum and starch were competing for the water. In contrast, at high concentration, gum was concentrated in the composite system itself and only starch was competing for the water, which might have caused diminution in solid-like properties.

Log of dynamic rheology data was also subjected to linear regression, *K*′ and *K*″ or *n*′ or *n*″ were calculated as displayed in Equations (3) and (4) and are presented in Table 3 where *k* designates the intercept while *n* specifies the slope of the line for both *K*′ and *K*″ or *n*′ and *n*″, which represents *G*′ and *G*″:

(1) $$G^{\prime }=K^{\prime }\omega^{n}$$

log *G*′= log *K*′+ *n*′ log ω (2)

*K*′ and *K*″ represents the consistency index based on *G*′ and *G*″, respectively, while *n*′ and *n*″ represents flow behavior index based on *G*′ and *G*″ of the power law. According to Choi and Chang [33], the slope of true gel should be zero while for weak gel it should be positive. In our case, the *K*′ value was higher than *K*″ for all samples and was increased almost two-folds with inclusion of xanthan, cress seed, fenugreek and okra at 0.5%. In contrast, they were decreased with gum arabic and flaxseed gums regardless of their concentration and also with higher levels (2.0%) of cress seed, fenugreek and okra as compared to the control. Furthermore, they were reduced as the concentration of gums increased from 0.5–2.0%, except xanthan enlightening that elastic character of the chickpea starch was reduced as the level of incorporated gum was increased and could be ascribed to the disruption of amylose network, which is the main constructor of the gel formation.

*K*′ and *K*″ represents the consistency index based on *G*′ and *G*″, *n*′ and *n*″ flow behavior index based on *G*′ and *G*″, R^2^ is the coefficient of determination. The results showed that slopes of log *G*′ and log *G*″ for all samples were positive, which gives the characteristics of typical weak gels, with R^2^ in the range of (0.54–0.92). The slopes of *G*″ for all samples were two–three-fold higher than the slopes of *G*′. The incorporation of gums into chickpea starch increased the *n*′ values, which showed the higher susceptibility of the chickpea starch gels to the applied stress and reduction in the viscous character with the addition of gums. The present results were in agreement with the previous researches of Choi and Chang [33] and Shaari et al. [34], who evaluated the effect of galactomannan on buckwheat starch and xanthan, carrageenan and pectin on corn starch, found that *n*′ was increased as a function of gum. In contrast, *n*″ values behaved differently with the incorporation of various gums in the starch.

## 4. Flow Behavior and Temperature Dependency

The power law was used to illustrate the difference in the rheological properties of starch gum blends as a function of variable shear rate. The model is widely used to explain the non-Newtonian liquids flowing properties both theoretically and practically. Fluids having flow behavior index 1.0 are considered Newtonian fluids, while those with less than 1.0 are called non-Newtonian or pseudoplastic. Parameters of power law used to describe steady flow behavior are summarized in Table 4. The data were well-fitted in the power law as indicated by the high values of coefficient of determination (0.98–0.99) as presented in Table 4. All blends had non-Newtonian shear thinning behavior (*n* < 1). Plain chickpea starch exhibited the highest flow behavior index (0.40) at 25 °C, which was reduced with the additions of gums regardless of their type. It was attributed to the large extent of structural breakdown during shearing due to the presence of gum. Singh et al. [30] reported similar findings with gum arabic and tapioca starch. However, no considerable reduction in flow behavior index was perceived at low levels of flaxseed and fenugreek. Similar results were reported by Choi and Yoo [35], in that the flow behavior index of the guar gum and sweet potato starch blend were less than control but did not vary much with increasing gum concentration up to 0.6%. Xanthan and starch blends possessed the lowest values, i.e., 0.34 and 0.32, at 25 °C, suggesting that they exhibited more pseudo-plastic shear thinning behavior. Although all gums exhibited a decrease in the flow behavior index values, xanthan had a pronounced effect because of its high molecular weight, rod-like conformation and more rigidity [36]. Previously, similar findings were reported by different authors with the addition of xanthan in maize, potato, rice and corn starch [34]. A substantial increase in the flow behavior index (*n*) was observed with increasing the temperature. This outcome clearly indicates a less pseudoplastic behavior of gels at higher temperatures.

Consistency coefficient (*K*) was used as an indicator of gel viscosity. Higher values indicated the high viscosity of the composite system and vice versa [37]. The value of *K* was amplified by the incorporation of gums (regardless of their type) except fenugreek. These results were consistent with Ji et al. [31], who reported a synergetic effect of gums on starch viscosity. However, there was remarkable increase (10.7 Pa·s) at higher xanthan concentration. This could be due to the thickening effect of xanthan gum which was noticed in the RVA experiments Wang et al. [38]. Considerable reduction in *K* was also noticed with increasing the temperature which could be attributed to the less pseudo plasticity (*n*) with increasing temperature [39].

The Arrhenius model was used to describe the effect of temperature on the rheological properties. It was crucial to evaluate the temperature effect, because heating at different temperatures could affect the food products containing gums and starches during processing. The effect of temperature was determined by fitting the data to Arrhenius equation. The log of consistency coefficient (*K*) was plotted against the inverse of temperature (Kelvin) and the activation energy was calculated from the slope of the line and results are listed in Table 5. The results revealed that there was a substantial increase in the activation energy of blends with the addition of gums except xanthan-starch blend. The highest value of activation energy (20,576 J/mol K^−1^) was observed in blend having 0.5% cress seed gum. However, the level of the influence was reliant on the type and concentration of gum. In contrast, decrease in the activation energy with xanthan was also observed, which indicated that the viscous properties were less dependent on temperature. Kim and Yoo [40] reported similar findings in rice starch with xanthan. As compared to other gums, xanthan exhibited less viscosity change with rising temperature concentration effect of temperature decreased [41]. Based on the activation energy values, it was concluded that a blend with xanthan was less temperature dependent compared to the control.

## 5. Texture Profile Analysis of Starch Gum Blends Gel

Texture profile analysis of gels from starch/gum blends stored overnight at room temperature is presented in Table 6. Hardness of fenugreek/starch blends were found significantly higher for both levels (8.58 N and 8.77 N) as compared to the control (8.23 N). The gum might have triggered competition for the water and reduction in amylose-amylose interactions [42]. Retrogradation comprises of transformation of amylose and amylopectin. Recrystallization of amylopectin and rearrangement of leached amylose could develop double helices and junction zones that ultimately lead to the establishment of organized structure of the starch gels during cooling [43]. However, the increase in gel hardness with addition of fenugreek gum did not agree with the previous studies. Brennan et al. [44] reported the reduced hardness of the wheat starch-fenugreek blend. This might be due to the difference in testing conditions and different nature of wheat starch granules and amylose contents. Furthermore, increase in hardness with the storage time was observed in potato and sweet potato starch with sodium alginate due to the rearrangement and recrystallization of amylose and amylopectin that enhance the gel networks during storage [45].

Arabic and cress seed gum blends were not found statistically different from the control at 0.5% and 2.0% gum concentration. However, hardness was found to decline with increasing gum concentration. This was attributed to the retardation effect of increased gum concentration on starch retrogradation, good water holding capacity and the gel properties [46]. Previously, different authors reported similar findings [44,46].

Hardness of flaxseed, okra and xanthan blends were significantly lower at both levels of gum except for flaxseed at 2.0% gum (7.99 N), when compared to the control (8.23 N). These observations indicated that these gums had ability to inhibit retrogradation of starch as shown by decrease in hardness compared to the control. This could be attributed to the hindering of network formation by the association between gums and leached amylose molecules [47]. The decrease in hardness with the addition of flaxseed, okra and xanthan are in line with the previous studies that hardness decreased with the addition of gums [48]. Springiness of the gel represents retrieval from distortion. It was significantly increased with xanthan, while decreased with fenugreek at both levels of gums (9.73 and 9.63 N). Reduction in the springiness of fenugreek resulted in slow polymer aggregate formation due to presence of gum causing more viscous region with less springiness [23]. Cohesiveness was significantly higher for okra, flaxseed and xanthan blends. A significant increase in adhesiveness was observed in all blends except fenugreek, which was statistically similar to the control.

## 6. Freeze–Thaw Stability of Starch Gum Blends Gel

The syneresis was determined to evaluate the capability of starch gum blends to tolerate undesirable physical changes that occurs by repeated freezing and thawing during storage and transportation. In freeze–thawed gel, it is mainly caused by retrogradation of amylose that results in the removal of water from the gel [49]. The effect of gums on the syneresis from chickpea starch gels at different levels is presented in Table 7. There was a significant increase in % syneresis in chickpea starch with okra during first and second thaw cycles, but statistically the same as the control during the last cycle. In contrast, it was decreased with xanthan during all three freeze thaw cycles.

The total % syneresis was significantly decreased with xanthan and cress seed, regardless of their concentration and with higher levels of gum arabic and fenugreek. This could be attributed to reduction in amylose degradation and increase in paste viscosity or because of the interaction between leached amylose and the gum [42]. Furthermore, these gums prevent the formation of spongy structure, which causes an increase in the amylose retrogradation. These finding was positively related to the previous findings where xanthan reduced % syneresis (0.85 and 1.0%) in corn starch [50]. In contrast, it was increased with okra irrespective of their concentration as well as with low levels of fenugreek and flaxseed. It might be because gum molecules were gathered and became less effectual, causing amylose to retrograde [23]. Syneresis was decreased as the concentration of gum was increased except for okra. It was attributed to the binding of water molecules with the gums or interaction between the gum and amylose [23,42].

## 7. Differential Scanning Calorimetry

Thermal properties like onset temperature (T_o_), peak temperature (T_p_) and enthalpy of gelatinization (ΔH) of plain and starch-gum blends are summarized in Table 8. Replacement of gums at both levels significantly affected the melting characteristics of chickpea starch. The onset and peak temperature varied as a function of gum type and their concentration. However, they were increased significantly with fenugreek irrespective of their concentration and with higher concentrations of other gums. These findings were in accordance with the pasting temperature determined through RVA experiment as shown in Table 1 except for the 2.0% xanthan gum. It was noticed that the onset temperature of blends measured by DSC were relatively lower than the pasting temperature measured by the RVA (Table 1) which indicates that the melting process leads to early increase in viscosity. It can be ascribed to the properties measured by two different methods. RVA measures gelatinization as an increase in the viscosity with torque, while DSC measures the energy required to melt starch crystals, as demonstrated by an endothermic peak. Furthermore, the presence of more starch decreases the pasting temperature, while at low level of moisture, the plasticizing effect of water was reduced, which resulted in a high melting temperature, as seen in DSC. The onset of starch gelatinization and starch swelling were delayed by the addition of gums at 2% level. These results were comparable with the previous findings, where the addition of xanthan, okra, flaxseed and sodium alginate elevated the gelatinization temperature of rice, maize, tuber (potato, sweet potato) and legume (chickpea and Turkish bean) starches [23,45,46,47]. The inclusion of gums might have delayed water migration into the starch due to the competition between gums and starch granules for water [43]. Another probable reason could be that gums (having higher water holding capacity) incorporation to the starch had reduced the available starch as compared to control [5].

Enthalpy of gelatinization (ΔH) increased significantly with flaxseed at 0.5% gum concentration and with 2.0% flaxseed, fenugreek and cress seed gums. Flaxseed exhibited the highest enthalpy (14.63 and 14.81 J/g) at both levels as compared to the control (13.25 J/g). The possible explanation for this significant increase in enthalpy could be the delay of water migration towards starch granule during gelatinization due to the hydrophilic behavior of gums, which resulted in more energy required for the process [43]. These findings were in agreement with the previous experiments where enthalpy was increased for maize and wheat/potato starch by the addition of flaxseed, pectin and konjac galactomannan [5,43]. However, it was decreased significantly with gum arabic and xanthan gum at both levels. Okra blends were not statistically different from the control. This was in accordance with the previous findings that gum inclusion in starch decreases the enthalpy [45,47]. The prominent decrease in enthalpy could be attributed to the strong hydrogen bonding between xanthan and leached amylose thus restricting the internal association between amylose and amylopectin [47]. The lower setback value of starch substituting xanthan gum measured through RVA experiments also supports the theory. The decrease is also associated with the limited mobility of amylopectin chains in the presence of gums, which also competed with amylose and amylopectin for the available water [18].

## 8. Conclusions

Pasting, rheological, textural and thermal properties of chickpea starch were greatly influenced by the incorporation of various gums. Peak viscosity of plain starch was increased in the presence of fenugreek and xanthan gum and these blends could be used in gluten- free bread formulations for better development of texture. The other gums have resulted in lower peak viscosities of starch blends. Starch blends with low viscosity could be used in confectionary products, weaning foods or as protective coating in different food applications. Enthalpy of gelatinization with flaxseed, cress seed and okra was increased while it was decreased in the presence of gum arabic and xanthan. Syneresis from the gels was decreased in the presence of cress seed and xanthan; therefore, these gums can be used in frozen products to improve their freeze thaw stability. All the chickpea starch/gum blends exhibited predominately elastic behavior (*G*′ > *G*″). Substantial increase in flow behavior index (*n*) was observed with increasing temperature. This outcome clearly indicates a less pseudoplastic behavior at higher temperatures. Although, all gums had represented non-Newtonian behavior with chickpea starch but fenugreek and flaxseed more stable (less structural breakdown during shearing) than other gums. All types of gum, except xanthan, required the highest amount of activation energy, which indicates that viscous properties were more dependent on temperature.

## 9. Material and Methods

### 9.1. Materials

Chickpea starch was extracted from chickpea grains purchased from the local market of Riyadh. Grains were cleaned to remove stones and extraneous material. Qualikems Fine Chem Pvt. Ltd. India, supplied commercial gum arabic and xanthan gums. Garden cress seeds, fenugreek seeds, flaxseeds and okra pods were also obtained from the local supermarket for gum extraction. All types of grains were cleaned to remove dirt, dust and broken seeds. Okra pods were washed and cut into halves and seeds were removed.

### 9.2. Methods

#### Isolation of Chickpea Starch

Chickpea starch was extracted according to the method of Alamri et al. [51]. Chickpea grains were soaked overnight in distilled water at a weight ratio of 1:2 and blended in household heavy duty blender for 2 min to make fine slurry. Slurry was filtered through muslin cloth. Filtrate was centrifuged at 2000× *g* for 10 min at 25 °C. Supernatant was discarded and the top dark layer containing protein material was removed from the precipitate. The white material was re-suspended in distilled water and re-three times following the above mentioned procedure. The obtained starch was mixed with acetone and air-dried at low temperature (40 °C). The dried starch was ground to fine powder in house hold blender, sieved and stored at refrigeration temperature in air tight jars until further use.

### 9.3. Extraction of Gums

#### Garden Cress Seed Gum

Cress seed gum was extracted according the method followed by Karazhiyan et al. [52]. Cleaned seeds (50 g) were soaked in 1 L of distilled water for 3 h under continuous stirring. Swollen seeds were squeezed with muslin cloth to collect the filtrate. Filtrate was freeze-dried, ground to fine powder and stored at 4 °C in airtight glass bottle.

### 9.4. Fenugreek Gum

For the extraction of fenugreek gum, (75 g) of cleaned seeds were soaked overnight in 1 L of distilled water under continuous stirring. Seeds were separated through filtration by muslin cloth. Filtrate was mixed with excess of ethanol (1:2) to precipitate the extracted gum. Mixing of ethanol was continued until precipitated gum was obtained. Precipitated gum was freeze-dried, ground to fine powder and stored at 4 °C in airtight glass bottle [53].

### 9.5. Flaxseed Gum

Flaxseed gum extraction was done according to Qian et al. [54]. Cleaned flaxseeds (125 g) were soaked overnight in 1 L of distilled water under continuous stirring. Flaxseeds were separated by filtration through muslin cloth. The filtrate was centrifuged to remove the insoluble particles. To precipitate the extracted gum, one volume of 100% ethanol was mixed with the supernatant and recovered by the centrifugation. Extracted gum was freeze dried, ground to fine powder and stored at 4 °C in glass bottle.

### 9.6. Okra Gum

Okra gum was extracted according to Alamri et al. [23]. Fresh okra pods were cut into halves and seeds were removed. Seedless okra pods halves (100 g) were blended in 500 mL 0.05 M NaOH for 5 min in a household blender. The blended mixture was centrifuged at 2000 *g* to separate the supernatant. The pH of supernatant was adjusted to 7, freeze dried, ground to fine powder and stored at 4 °C in airtight glass bottle.

### 9.7. Preparation of Starch Gum Blends

Starch/gum blends (*w*/*w*) in triplicate were prepared by replacing 0.5 and 2% of starch with each gum (gum arabic, xanthan, garden cress seed, flaxseed, fenugreek and okra). Wet mixing method was used for the preparation of blends. Slurry of starch and gum was prepared with distilled water, mixed thoroughly and freeze-dried. Plain chickpea starch was used as a control throughout the studies.

### 9.8. Pasting Properties of Starch Gum Blends

It was determined by using Rapid Visco Analyzer (Newport Scientific, Sydney, Australia) according to the method of Alamri, Mohamed and Hussain [20]. Sample (3 g) at 14% moisture basis was weighed in RVA aluminum canister and distill water was added to make slurry of total weight of 28 g. Paddle speed was kept at 960 rpm for initial 10 s and then decreased to 160 rpm throughout the experiment. A programmed heating and cooling cycle was designed. Resultant slurry was kept at 50 °C for 30 s, heated from 50 °C to 95 °C in 4.40 min at rate of 10.23 °C/min, kept at 95 °C for 4 min. Afterwards, it was cooled from 95 °C to 50 °C in 4.40 min at rate of 10.23 °C/min, and finally maintained at 50 °C for 2 min. All measurements were done in triplicate and data was processed by Thermocline software (Newport Scientific, Sydney, Australia).

### 9.9. Dynamic Rheology, Steady Flow Behavior and Temperature Dependency

The dynamic viscoelastic properties of starch pastes were determined by using TA (TA Instruments Inc., New Castle, DE, USA), Discovery Hybrid Rheometer (HR-1) installed with cone (2°) plate geometry (40 mm) in diameter. The gap between cone and plate was set as 100 µm. Starch/gum blend samples (5% *w*/*v*) were cooked in RVA by using a same method as mentioned above. Samples were transferred to plate and rheometer was calibrated at 25 °C for 1 min. Excess of sample was wiped off with spatula and geometry gap was adjusted to 100 µm. Dynamic shear data was obtained at frequency sweeps ranging from 0.1–100 rad/s at 5% constant strain at 25 °C. Experimental data were collected and rheological parameters were calculated using the instrument software. The storage moduli (*G*′), loss moduli (*G*″) and dynamic mechanical loss tangent (tanδ = *G*″/*G*′) were recorded. Shear rate versus shear stress data was also recorded at 25, 45 and 65 °C and activation energy was determined by using the Arrhenius Equation model (1). The shear stress versus shear rate dependence was estimated by fitting the data with power law (2), the most popular model to explain the steady shear flow behavior.

(ln*μa* = ln*μ^o^* + *Ea*/RT) (3)

*µa* = apparent viscosity,

*µ_o_* = frequency factor,

R = gas constant,

*Ea*= energy of activation,

T = absolute temperature (K)

Frequency factor is a constant that relates the frequency of the colliding molecules with enough energy to start a reaction, and it varies from reaction to reaction.

The reciprocals of temperatures (25, 45 and 65 °C converted to Kelvin as 298.15, 318.15 and 338.15, respectively) were plotted against the natural log of *K* values presented in Table 4. After applying the linear regression, slope was multiplied with universal gas constant (R = 8.314 j/mol·K) to get the value of Ea, while inverse log of intercept was considered as *µ_o_* = frequency factor:

(4) $$T={K_{\gamma }}^{n}$$

where T = shear stress (Pa·s), K = consistency coefficient (Pa·s), γ = shear rate (s^−1^) and n = flow behavior index (dimensionless).

### 9.10. Texture Profile Analysis of Starch Gum Blends Gel

Textural properties of starch gels were determined by using Brookfield Texture Analyzer model CT3 (Brookfield Engineering Laboratories, Inc., Middleboro, MA, USA) according to the method of Alamri et al. [55]. The starch gels obtained from rapid visco analyzer were kept in aluminum canisters (65 mm in height and 36 mm diameter) and stored overnight at room temperature. The gels were compressed with cylindrical probe (35 mm high and 12.7 mm diameter) in two penetration cycles with a speed of 0.5 mm/second up to 10 mm. Hardness, cohesiveness, springiness and adhesiveness were directly noted. Gumminess was calculated as a product of hardness and cohesiveness, while chewiness was calculated as a product of gumminess and springiness.

### 9.11. Freeze–Thaw Stability of Starch Gum Blends Gel

Freeze–thaw stability of the gels was determined as described by Alamri et al. [23], with slight modification. RVA cooked starch gels were transferred to centrifuge tubes and stored at −20 °C in the freezer. Tubes were removed from the freezer on 7th day and kept in hot water bath for 30 min at 50 °C. The tubes were centrifuged at 3000× *g* for 15 min and the water that separated from the gels was calculated and expressed as % syneresis relative to weight of original sample. This was called as one thaw cycle. The tubes were then restored in freezer for another 7 days for a 2nd thaw cycle, and the % syneresis was calculated. This process was repeated again for a 3rd cycle. Percent syneresis for each cycle was reported separately, and for a total of three freeze- thaw cycles.

### 9.12. Differential Scanning Calorimetry

Thermal properties of starch blends were determined by using TA instruments (TA Instruments Inc., New Castle, DE, USA), DSC, Q 2000 according to the method followed by [53]. Starch/gum blends (5–10 mg) were weighed in aluminum pans and 60–80% distilled water was added. Pans were hermetically sealed and allowed to equilibrate at room temperature for 2 h. Thermal scanning was done from 25–120 °C at 10 °C heating rate. Empty pan was used as a reference cell. Thermal transitions were measured as enthalpy ∆H (J/g), onset temperature (T_o_) and peak temperature (T_p_) using Universal Analysis Software.

## 10. Statistical Analysis

All readings were collected in triplicate. Data was analyzed using one-way ANOVA method. This analysis allowed us to detect the significant effect of gums on chickpea starch. Duncan’s Multiple Range (DMR) test at sig ≤ 0.05 was used to compare means using SPSS (IBM Statistical Analysis Version 21).

## Figures and Tables

**Table 1:** Effect of gums on pasting properties of chickpea starch.

Parameter	Gum %	Control	Arabic	Xanthan	Cress Seed	Fenugreek	Flaxseed	Okra
Peak Viscosity (cP) ^a^	0.5	3807 ± 22 ^d^	3496 ± 14 ^f^	4256 ± 16 ^a^	3928 ± 21 ^c^	4040 ± 79 ^b^	3703 ± 33 ^e^	3370 ± 03 ^g^
	2.0	3807 ± 22 ^c^	2804 ± 16 ^g^	5611 ± 109 ^a^	3257 ± 14 ^e^	4311 ± 95 ^b^	3140 ± 15 ^f^	3528 ± 37 ^d^
Breakdown Viscosity (cP)	0.5	1350 ± 47 ^a^	1153 ± 44 ^b,c^	1069 ± 31 ^c^	1354 ± 79 ^a^	1270 ± 118 ^a,b^	1211 ± 40 ^b^	1157 ± 14 ^b,c^
	2.0	1350 ± 47 ^a^	0789 ± 22 ^c^	1419 ± 09 ^a^	1085 ± 11 ^b^	1333 ± 51 ^a^	1035 ± 11 ^b^	1073 ± 116 ^b^
Final Viscosity (cP)	0.5	6146 ± 109 ^b,c^	5688 ± 38 ^d^	6317 ± 102 ^a^	6067 ± 22 ^c^	6207 ± 72 ^a,b^	5620 ± 43 ^d^	5115 ± 14 ^e^
	2.0	6146 ± 109 ^c^	4958 ± 55 ^d^	6760 ± 190 ^a^	5039 ± 39 ^d^	6330 ± 51 ^b^	4757 ± 07 ^e^	4935 ± 17 ^d^
Setback (cP)	0.5	3691 ± 133 ^a^	3344 ± 94 ^b,c^	3140 ± 120 ^c^	3503 ± 68 ^a,b^	3417 ± 269 ^b^	3146 ± 32 ^c^	2899 ± 05 ^d^
	2.0	3691 ± 133 ^a^	2943 ± 19 ^c^	2550 ± 78 ^d,e^	2867 ± 33 ^c^	3351 ± 37 ^b^	2671 ± 24 ^d^	2461 ± 87 ^e^
Pasting Temperature (°C)	0.5	69.83 ± 0.4 ^b^	70.61 ± 0.4 ^a^	70.56 ± 0.4 ^a^	70.68 ± 0.4 ^a^	70.56 ± 0.5 ^a^	71.03 ± 0.1 ^a^	71.16 ± 0.1 ^a^
	2.0	69.83 ± 0.4 ^d^	70.61 ± 0.4 ^c,d^	68.11 ± 1.0 ^e^	72.10 ± 0.4 ^b^	71.73 ± 0.2 ^b^	71.30 ± 0.4 ^b,c^	73.45 ± 0.1 ^a^

Mean values with different superscripts within the same rows are significantly (*p* < 0.05) different. ^a^ cP = Centipoise.

**Table 2:** Effect of gums on calculated, RVA-measured setback and % change in setback.

Parameters	Calculated Setback Value Due to Starch Replacement		RVA Measured Values		Difference ^a^ Due to Gum		% Reduction ^b^ in Setback Due to Gum	
Gum %	0.5	2.0	0.5	2.0	0.5	2.0	0.5	2.0
Control	3691	3691	3691	3691	−	−	−	−
Arabic	3672	3617	3344	2943	328	674	9	19
Xanthan	3672	3617	3140	2550	532	1067	14	30
Cress seed	3672	3617	3503	2867	169	750	05	21
Fenugreek	3672	3617	3417	3351	255	321	07	07
Flaxseed	3672	3617	3146	2671	526	946	14	26
Okra	3672	3617	2899	2461	773	1156	21	32

^a^ Difference = calculated value − Measured value; ^b^ Difference/calculated value × 100.

**Table 3:** Effect of gums on *K*′, *K*″, *n*″ and *n*″ as determined from Equations (1) and (2).

	Gum %	*K*′	*n*′	R^2^	*K*″	*n*″	R^2^
Control	−	2.600 ^b^	0.101 ^b^	0.72 ^a^	1.209 ^c^	0.277 ^c^	0.89 ^a^
Arabic	0.5	2.498 ^c^	0.106 ^b^	0.70 ^a^	1.043 ^d^	0.274 ^c^	0.87 ^b^
Xanthan	0.5	2.613 ^b^	0.140 ^a^	0.73 ^a^	1.222 ^c^	0.320 ^a^	0.91 ^a^
Cress seed	0.5	2.682 ^b^	0.114 ^b^	0.63 ^b^	1.319 ^b^	0.248 ^d^	0.82 ^c^
Fenugreek	0.5	2.641 ^b^	0.111 ^b^	0.64 ^b^	1.212 ^c^	0.292 ^b^	0.92 ^a^
Flaxseed	0.5	2.543 ^c^	0.111 ^b^	0.64 ^b^	1.085 ^d^	0.278 ^c^	0.86 ^b^
Okra	0.5	2.857 ^a^	0.115 ^b^	0.54 ^c^	1.469 ^a^	0.271 ^c^	0.71 ^d^
Control	−	2.600 ^a^	0.101 ^b^	0.72 ^b^	1.209 ^b^	0.277 ^b^	0.89 ^b^
Arabic	2.0	2.428 ^c^	0.103 ^d^	0.70 ^b,c^	0.943 ^d^	0.266 ^b^	0.77 ^d^
Xanthan	2.0	2.424 ^c^	0.136 ^a^	0.79 ^a^	1.559 ^a^	0.199 ^d^	0.89 ^b^
Cress seed	2.0	2.548 ^b^	0.109 ^d^	0.68 ^c^	1.114 ^c^	0.254 ^b,c^	0.88 ^b^
Fenugreek	2.0	2.405 ^c^	0.108 ^d^	0.68 ^c^	0.954 ^d^	0.312 ^a^	0.92 ^a^
Flaxseed	2.0	2.556 ^b^	0.132 ^a^	0.63 ^d^	1.059 ^d^	0.319 ^a^	0.84 ^c^
Okra	2.0	2.598 ^a^	0.118 ^d^	0.60 ^e^	1.239 ^b^	0.239 ^c^	0.87 ^b^

Mean values with different superscripts within the same rows are significantly (*p* < 0.05) different.

**Table 4:** Effect of gums at different temperature on consistency coefficient (*K*) and flow behavior index (*n*) of chickpea starch measured at 25, 45 and 65 °C.

Temperature	Parameter	Gum %	Control	Arabic	Xanthan	Cress Seed	Fenugreek	Flaxseed	Okra
25 °C	^1^ *K*	0.5	5.86 ^d^	6.07 ^c^	7.56 ^a^	7.09 ^b^	5.83 ^d^	5.98 ^c,d^	6.14 ^c^
		2.0	5.86 ^e^	6.56 ^c^	10.7 ^a^	6.26 ^d^	5.88 ^e^	6.22 ^d^	7.07 ^b^
	^2^ *n*	0.5	0.40 ^a^	0.36 ^b^	0.34 ^c^	0.35 ^b,c^	0.39 ^a^	0.39 ^a^	0.36 ^b^
		2.0	0.40 ^a^	0.35 ^c^	0.32 ^d^	0.36 ^b,c^	0.36 ^b,c^	0.37 ^b^	0.34 ^c,d^
	R^2^	0.5	0.99 ^a^	0.99 ^a^	0.99 ^a^	0.98 ^a^	0.99 ^a^	0.99 ^a^	0.99 ^a^
		2.0	0.99 ^a^	0.99 ^a^	0.99 ^a^	0.99 ^a^	0.99 ^a^	0.99 ^a^	0.98 ^a^
45 °C	*K*	0.5	5.40 ^c^	4.90 ^e^	6.32 ^a^	5.78 ^b^	5.29 ^d^	5.40 ^c^	5.27 ^d^
		2.0	5.40 ^c^	5.19 ^c^	9.01 ^a^	6.04 ^b^	4.26 ^d^	5.04 ^c^	6.16 ^b^
	*n*	0.5	0.40 ^b^	0.42 ^a^	0.38 ^c^	0.36 ^d^	0.39 ^b,c^	0.40 ^b^	0.36 ^d^
		2.0	0.40 ^a^	0.41 ^a^	0.34 ^d^	0.38 ^b^	0.40 ^a^	0.40 ^a^	0.36 ^c^
	R^2^	0.5	0.99 ^a^	0.98 ^a^	0.99 ^a^	0.98 ^a^	0.99 ^a^	0.99 ^a^	0.98 ^a^
		2.0	0.99 ^a^	0.99 ^a^	0.99 ^a^	0.99 ^a^	0.99 ^a^	0.99 ^a^	0.99 ^a^
65 °C	*K*	0.5	2.90 ^b^	2.77 ^d,e^	3.79 ^a^	2.92 ^b^	2.72 ^e^	2.80 ^c^	2.64 ^f^
		2.0	2.90 ^c,d^	2.72 ^e^	7.80 ^a^	2.99 ^c^	2.84 ^d^	2.84 ^d^	3.43 ^b^
	*n*	0.5	0.48 ^b^	0.48 ^b^	0.46 ^c^	0.46 ^c^	0.49 ^a^	0.49 ^a^	0.47 ^c^
		2.0	0.48 ^a^	0.49 ^a^	0.36 ^d^	0.46 ^b^	0.46 ^b^	0.48 ^a^	0.43 ^c^
	R^2^	0.5	0.99 ^a^	0.99 ^a^	0.99 ^a^	0.99 ^a^	0.99 ^a^	0.99 ^a^	0.99 ^a^
		2.0	0.99 ^a^	0.99 ^a^	0.99 ^a^	0.99 ^a^	0.99 ^a^	0.99 ^a^	0.99 ^a^

Mean values with different superscripts within the same rows are significantly (*p* < 0.05) different. ^1^ *K =* (Pa·s) indices are obtained by fitting the data to power law Τ = *Kγ* · *^n^* and ^2^ *n* = flow behavior index (dimensionless). This table was used for the Arrhenius equation.

**Table 5:** Effect of gums on Arrhenius-type equation parameters of chickpea starch.

Parameters	*µ*_o_ (Pa s^n^) ^A^		*Ea* (J/mol K^−1^) ^B^		R^2^	
Gum %	0.5	2.0	0.5	2.0	0.5	2.0
Control	1.04 × 10^−4 e^	1.04 × 10^−4 e^	14,485 ^d^	14,485 ^d^	0.80	0.80
Arabic	2.02 × 10^−5 d^	3.93 × 10^−6 c^	16,262 ^b,c^	18,243 ^a^	0.91	0.91
Xanthan	2.04 × 10^−4 d^	4.95 × 10^−1 b^	14,305 ^d^	6628 ^e^	0.91	0.99
Cress seed	5.74 × 10^−7 b^	6.69 × 10^−5 a^	20,576 ^a^	15,171 ^c^	0.87	0.76
Fenugreek	3.33 × 10^−5 c^	4.59 × 10^−5 b^	15,706 ^c^	15,194 ^c^	0.82	0.99
Flaxseed	3.75 × 10^−5 c^	2.17 × 10^−5 d^	15,637 ^c^	16,247 ^b^	0.82	0.91
Okra	7.52 × 10^−6 a^	1.01 × 10^−4 e^	17,428 ^b^	14,941 ^d^	0.86	0.87

Mean values with different superscripts within the same rows are significantly (*p* < 0.05) different. ^A^ *μ*_o_ (Pa s^n^) = is the frequency factor at a reference temperature (25, 45 and 65 °C); ^B^ *Ea* = activation energy (J/mol K^−1^) parameters were obtained by fitting experimental data to Arrhenius equation (ln*μ_a_* = ln*μ_o_* + *Ea*/RT).

**Table 6:** Effect of gums on the TPA for chickpea starch stored overnight at room temperature.

Parameters	Gum %	Control	Arabic	Xanthan	Cress Seed	Fenugreek	Flaxseed	Okra
Hardness (N)	0.5	8.23 ± 0.21 ^b^	8.31 ± 0.18 ^b^	7.47 ± 0.14 ^d^	8.29 ± 0.06 ^b^	8.58 ± 0.12 ^a^	7.93 ± 0.19 ^c^	7.82 ± 0.09 ^c^
	2.0	8.23 ± 0.21 ^b^	8.17 ± 0.12 ^b,c^	6.63 ± 0.15 ^d^	8.02 ± 0.13 ^b,c^	8.77 ± 0.09 ^a^	7.99 ± 0.19 ^b,c^	7.90 ± 0.17 ^c^
Cohesiveness	0.5	0.53 ± 0.02 ^b^	0.51 ± 0.01 ^b^	0.56 ± 0.02 ^a^	0.53 ± 0.01 ^b^	0.47 ± 0.02 ^c^	0.56 ± 0.02 ^a^	0.57 ± 0.01 ^a^
	2.0	0.53 ± 0.02 ^d,e^	0.51 ± 0.01 ^e^	0.61 ± 0.01 ^a^	0.54 ± 0.01 ^c,d^	0.45 ± 0.02 ^f^	0.57 ± 0.01 ^b^	0.55 ± 0.01 ^b,c^
Springiness (mm)	0.5	9.90 ± 0.00 ^b,c^	9.80 ± 0.00 ^c,d^	10.17 ± 0.06 ^a^	9.80 ± 0.10 ^c,d^	9.73 ± 0.06 ^d^	9.97 ± 0.06 ^b^	9.97 ± 0.15 ^b^
	2.0	9.90 ± 0.00 ^c^	10.07 ± 0.06 ^b^	10.27 ± 0.06 ^a^	9.87 ± 0.06 ^c^	9.63 ± 0.06 ^d^	10.0 ± 0.17 ^b,c^	9.93 ± 0.06 ^b,c^
Adhesiveness (mJ)	0.5	0.03 ± 0.06 ^c^	0.13 ± 0.06 ^a,b^	0.17 ± 0.06 ^a,b^	0.17 ± 0.06 ^a,b^	0.10 ± 0.00 ^b,c^	0.17 ± 0.06 ^a,b^	0.20 ± 0.00 ^a^
	2.0	0.03 ± 0.06 ^c^	0.23 ± 0.06 ^a^	0.13 ± 0.06 ^a,b^	0.17 ± 0.06 ^a,b^	0.10 ± 0.00 ^b,c^	0.13 ± 0.06 ^a,b^	0.17 ± 0.06 ^a,b^
Gumminess N	0.5	4.34 ± 0.16 ^a,b^	4.21 ± 0.11 ^b,c^	4.21 ± 0.08 ^b,c^	4.40 ± 0.07 ^a,b^	4.03 ± 0.14 ^c^	4.47 ± 0.03 ^a^	4.45 ± 0.08 ^a^
	2.0	4.34 ± 0.16 ^b^	4.19 ± 0.04 ^b,c^	4.04 ± 0.17 ^c^	4.33 ± 0.14 ^b^	3.98 ± 0.19 ^c^	4.55 ± 0.09 ^a^	4.37 ± 0.05 ^a,b^
Chewiness (N.mm)	0.5	42.93 ± 1.56 ^a,b^	41.24 ± 1.06 ^b,c^	42.78 ± 1.02 ^a,b^	43.07 ± 0.86 ^a,b^	39.33 ± 1.46 ^c^	44.52 ± 0.17 ^a^	44.88 ± 1.45 ^a^
	2.0	42.93 ± 1.56 ^b^	42.22 ± 0.63 ^b^	41.51 ± 0.92 ^b^	42.73 ± 1.59 ^b^	38.30 ± 2.05 ^c^	45.52 ± 0.18 ^a^	43.44 ± 0.57 ^a b^

Mean values with different superscripts within the same rows are significantly (*p* < 0.05) different.

**Table 7:** Effect of gums on the % syneresis of chickpea starch.

Days	7th Day		14th Day		21st Day		Total	
Gum %	0.5	2.0	0.5	2.0	0.5	2.0	0.5	2.0
Control	2.72 ± 0.11 ^c,d^	2.72 ± 0.49 ^b^	1.66 ± 0.35 ^b^	1.66 ± 0.54 ^b^	1.51 ± 0.16 ^a^	1.51 ± 0.16 ^a^	5.90 ± 0.39 ^c^	5.90 ± 0.39 ^b^
Arabic	2.95 ± 0.73 ^b,c,d^	2.24 ± 0.23 ^c^	2.35 ± 0.45 ^a^	1.74 ± 0.18 ^b^	0.50 ± 0.26 ^c^	1.30 ± 0.22 ^a,b^	5.81 ± 0.09 ^c^	5.29 ± 0.19 ^c,d^
Xanthan	2.46 ± 0.62 ^d^	1.32 ± 0.38 ^d^	0.60 ± 0.11 ^c^	0.34 ± 0.11 ^c^	0.66 ± 0.30 ^c^	0.87 ± 0.09 ^c^	3.64 ± 0.23 ^e^	2.54 ± 0.34 ^e^
Cress seed	2.66 ± 0.30 ^c,d^	4.05 ± 0.24 ^a^	1.77 ± 0.05 ^b^	0.65 ± 0.28 ^c^	0.58 ± 0.21 ^c^	0.51 ± 0.20 ^d^	5.02 ± 0.12 ^d^	5.22 ± 0.61 ^c,d^
Fenugreek	3.60 ± 0.26 ^b^	2.79 ± 0.02 ^b^	1.63 ± 0.31 ^b^	1.66 ± 0.02 ^b^	1.43 ± 0.26 ^a^	0.49 ± 0.09 ^d^	6.67 ± 0.24 ^b^	4.94 ± 0.10 ^d^
Flaxseed	3.40 ± 0.32 ^b,c^	2.68 ± 0.13 ^b^	2.38 ± 0.11 ^a^	1.79 ± 0.39 ^b^	0.81 ± 0.18 ^b,c^	1.12 ± 0.28 ^b,c^	6.60 ± 0.08 ^b^	5.59 ± 0.17 ^b,c^
Okra	5.34 ± 0.37 ^a^	3.82 ± 0.30 ^a^	2.52 ± 0.34 ^a^	2.59 ± 0.58 ^a^	1.16 ± 0.21 ^a,b^	0.45 ± 0.12 ^d^	9.03 ± 0.35 ^a^	6.87 ± 0.26 ^a^

Mean values with different superscripts within the same column are significantly (*p* < 0.05) different.

**Table 8:** Effect of gums on the DSC profile of chickpea starch.

Parameters	T_0_ (°C) ^A^		T_p_ (°C) ^B^		ΔH (J/g) ^C^	
Gum %	0.5	2.0	0.5	2.0	0.5	2.0
Control	54.71 ± 0.28 ^a,b^	54.71 ± 0.28 ^d^	63.10 ± 0.32 ^c^	63.10 ± 0.32 ^d^	13.25 ± 0.54 ^b^	13.25 ± 0.54 ^b^
Arabic	55.10 ± 0.20 ^a,b^	55.71 ± 0.22 ^b^	63.33 ± 0.15 ^b,c^	63.64 ± 0.20 ^c^	11.99 ± 0.93 ^c^	11.70 ± 0.87 ^c^
Xanthan	55.19 ± 0.67 ^a,b^	55.44 ± 0.15 ^b,c^	63.59 ± 0.02 ^b,c^	64.41 ± 0.51 ^b^	04.24 ± 0.14 ^d^	04.00 ± 0.36 ^d^
Cress seed	54.60 ± 0.33 ^b^	55.03 ± 0.18 ^c,d^	63.83 ± 0.63 ^b^	64.32 ± 0.26 ^b^	13.91 ± 0.76 ^a,b^	14.74 ± 0.44 ^a^
Fenugreek	55.55 ± 0.62 ^a^	55.76 ± 0.40 ^b^	63.80 ± 0.17 ^b^	64.11 ± 0.24 ^b,c^	14.14 ± 0.05 ^a,b^	14.34 ± 0.18 ^a^
Flaxseed	54.96 ± 0.54 ^a,b^	55.48 ± 0.52 ^b,c^	63.58 ± 0.17 ^b,c^	64.02 ± 0.19 ^b,c^	14.63 ± 0.31 ^a^	14.81 ± 0.56 ^a^
Okra	54.89 ± 0.44 ^a,b^	56.51 ± 0.29 ^a^	64.53 ± 0.06 ^a^	65.58 ± 0.03 ^a^	13.42 ± 0.63 ^b^	13.19 ± 0.83 ^b^

Mean values with different superscripts within the same column are significantly (*p* < 0.05) different. ^A^ T_0_ = onset temperature; ^B^ T_p_ = peak temperature; ^C^ ΔH = enthalpy of starch crystal disassociation.
